# Anterior segment dysgenesis associated with Williams-Beuren syndrome: a case report and review of the literature

**DOI:** 10.1186/1471-2415-14-70

**Published:** 2014-05-21

**Authors:** Margarita G Todorova, Matthias C Grieshaber, Rafael JA Cámara, Peter Miny, Anja M Palmowski-Wolfe

**Affiliations:** 1 Department of Ophthalmology, University of Basel, Mittlere Strasse 91, Basel CH-4031, Switzerland; 2 Department of Medical Genetics, University of Basel, Basel, Switzerland

**Keywords:** Williams-Beuren syndrome, Anterior segment dysgenesis, Fluorescence in-situ hybridization, Microdeletion of chromosome 7q11.23, Peters' anomaly, Ultrasound biomicroscopy

## Abstract

**Background:**

Williams-Beuren syndrome is characterized by mild mental retardation, specific neurocognitive profile, hypercalcemia during infancy, distinctive facial features and cardiovascular diseases. We report on complete ophthalmologic, sonographic and genetic evaluation of a girl with a clinical phenotype of Williams-Beuren syndrome, associated with unilateral anterior segment dysgenesis and bilateral cleft of the soft and hard palate. These phenotypic features have not been linked to the haploinsufficiency of genes involved in the microdeletion.

**Case presentation:**

A term born girl presented at the initial examination with clouding of the right cornea. On ultrasound biomicroscopy the anterior chamber structures were difficult to differentiate, showing severe adhesions from the opacified cornea to the iris with a kerato-irido-lenticular contact to the remnant lens, a finding consistent with Peters' anomaly. Genetic analyses including FISH confirmed a loss of the critical region 7q11.23, usually associated with the typical Williams-Beuren syndrome. Microsatellite analysis showed a loss of about 2.36 Mb.

**Conclusions:**

A diagnosis of Williams-Beuren syndrome was made based on the microdeletion of 7q11.23. The unique features, including unilateral microphthalmia and anterior segment dysgenesis, were unlikely to be caused by the microdeletion. Arguments in favor of the latter are unilateral manifestation, as well as the fact that numerous patients with deletions of comparable or microscopically visible size have not shown similar manifestations.

## Background

Williams-Beuren syndrome (WBS, MIM #194050) is a clinically recognizable and recurrent condition associated with a variably-sized hemizygous deletion within band 11.23 of the long arm of chromosome 7, detectable by FISH [1,2].

The clinical phenotype of WBS includes mild mental retardation, specific neurocognitive profile, hypercalcemia during infancy, distinctive facial features and cardiovascular diseases. Facial features include prognathism, mild midfacial hypoplasia, as well as a nose with rounded upward turned tip and forward pointing nostrils, prominent lips and a geographic tongue. Cardiovascular diseases related to the syndrome are aortic hypoplasia, which occurs in almost 75% of patients, supravalvular aortic stenosis and peripheral pulmonary artery stenosis, atrial and ventricular septal defects [1-7].

Ocular features, reported as a hallmark of the WBS, are strabismus (29-78%) [8-10] with predominance of esotropia (52%) [10], especially the hereditary form of infantile esotropia (59%) [8], dissociated vertical deviation (37%) [11], oblique muscle dysfunction (53%) [11], amblyopia (32%) [12], cataract (1.97%) [10] and ptosis (1.32%) [10]. Additional ocular features of the syndrome are a stellate pattern of the anterior iris stroma (62-74%) [9,10] noted especially in individuals with a light colored iris, as well as retinal vascular tortuosity (22%) [10] and a situs inversus vasorum (15%) [9].

Approximately 28 transcribed genes have been mapped in the deleted 7q11.23 Williams-Beuren region, such as: elastin (*ELN*), replication factor C (*RFC*), LIM domain kinase 1 (*LIMK1*), general transcription factor 2 (*GTF21*), GTF21 repeat domain 1 (*GTF21RD1*), FK506 binding protein 6 (*FKBP6*) [13]. The best understood ophthalmic feature within the WBS deleted region is in connection with the hemizygosity of *ELN*[14].

Anterior segment dysgenesis (ASD) disorders may display a wide variety of developmental conditions affecting the cornea, iris, ciliary body, anterior chamber and lens. The condition could be isolated or related to a systemic disease. A number of genes have been linked to anterior segment morphogenesis, coding the migration of the neural crest cells, but also regulating mesenchymal cell differentiation of the anterior segment tissues [15,16]. For instance, a paired-like homeodomain transcription factor 2 (*PITX2*) is associated with Axenfeld-Rieger syndrome [17,18], iridogoniodysgenesis syndrome, and sporadic cases of Peters’ anomaly. A forkhead box C1 (*FOXC1*) is considered to be a cause for primary congenital glaucoma, autosomal dominant iridogoniodysgenesis anomaly and Axenfeld-Rieger anomaly [17,18]. A paired box gene 6 (*PAX6*) leads to ocular tissue arrest, such as in aniridia [19], and has also been associated with Peters' anomaly.

Hereby, we report the case of a 6-year-old girl with a clinical phenotype of WBS, associated with unilateral anterior segment dysgenesis and bilateral cleft of the soft and hard palate. These phenotypic features have not been linked so far to the haploinsufficiency of genes involved in the *WBS 7q11.23* microdeletion in other reports.

## Case presentation

A term born girl was referred shortly after birth for ophthalmic examination due to clouding of the right cornea.

The findings at the initial examination included paracentral stromal opacity of the cornea, coloboma and transillumination of the iris of the right microphthalmic eye. Conjunctival scraping, blood cultures and serologic testing to rule out neonatal herpes simplex and cytomegalovirus showed no evidence of inflammation. A bulla, 3 mm in diameter, with an apical protrusion was seen within the corneal opacity. This was suspicious of a descemetocele due to severe keratoconus or corneal ectasia. On palpation, the intraocular pressure of the right eye was as low as of the left eye. The corneal clouding precluded visualization of the anterior chamber angle and the posterior segment. Bilaterally a diagnostic B-ultrasound of the posterior segment showed no pathology. The axial eye length, measured with A-scan, was 14.34 mm in the right eye and 18.06 mm in the left eye. Except for a stellate pattern of the iris, the anterior segment of the left eye showed no pathological findings.

On physical examination, the initial inspiratory stridor was explained as being related to prognathism and bilateral cleft palate. In addition, patent foramen ovale and patent ductus arteriosus were diagnosed on ultrasonography. Laboratory parameters including the level of serum-ionized calcium in infancy were normal (1.45 mmol/l).

Pregnancy history revealed a maternal intake of undetermined medications for 2–3 months during the pregnancy. The family history except for a maternal uncle with a cleft palate was otherwise unremarkable.

Due to the cleft palate, the prognathia, as well as the positive history of cleft palate in the family, a Pierre-Robin sequence was suspected initially. Chromosomal analysis at birth showed a normal *46, XX* karyotype without numerical or structural chromosome anomalies. Furthermore, the ophthalmological findings were not compatible with the Pierre-Robin sequence, and since dysmorphic features, as well as, muscular hypotonia and developmental delay gradually became more apparent, a Williams-Beuren phenotype was suspected. Fluorescence in-situ hybridization (FISH) was performed (Q Biogene, currently MP Biomedicals, probe number CP5155-DC) and confirmed a loss of the critical region *7q11.23* (WSCR). The deletion size was characterized further by molecular tools (microsatellite analysis, polymorphic microsatellite markers: D7S672, D7S489B, D7S2476, D7S1870, D7S489A, D7S2490, D7S2518, D7S2470, D7S669) and a loss of about 2.36 Mb was confirmed.

At the age of six months, the bilateral clefts of the soft and the hard palate were adapted surgically. The facial phenotype developed with a broad forehead, bitemporal narrowing, low nasal root, periorbital fullness, full cheeks and lips and wide opened mouth. Dysmorphic dental aberrations included agenesis, smaller size and aberrant shape of the teeth. The neurophysiologic profile showed a mild deficit in nonverbal spatial cognition, peculiar behavior, with expressive sociability, talkativeness, hypersensitivity to light and noise.

Ophthalmological follow up was performed on a regular basis. A penetrating keratoplasty or a tectonic graft were discussed but not performed due to the young age of the patient and the poor prognosis.

**Figure 1:**
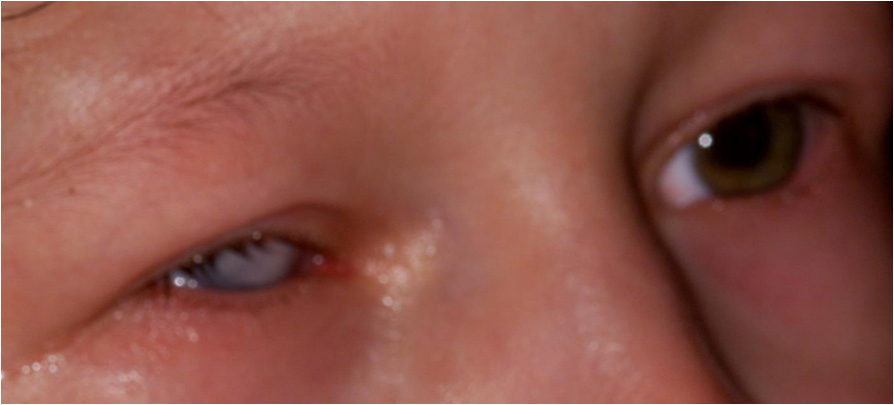
**Anterior segment photograph, left eye. **Figure 1 depicts an anterior segment photograph, presenting a central clouding and vascularized microcornea of the right eye; no pathological findings of the left eye were recorded.

At the age of six years her best-corrected distance/near vision was recorded as 20/20 in the left eye and no light perception in the right eye. A follow up ophthalmic examination under general anesthesia revealed a slightly small, vascularized cornea of the right eye with a diameter of 11.0 mm vertically and 10.5 mm horizontally (Figure 1). The intraocular pressure (measured by Perkins applanation tonometry) in the right eye was 11 mmHg and in the left eye – 8 mmHg. Ophthalmoscopy of the left eye showed a normal optic disc and a macula with a mottling of the pigment epithelium. Vascular tortuosity in the inferior quadrants of the posterior pole, noted in the previous examinations, was confirmed. In the right eye, the corneal clouding prevented gonioscopy, retinoscopy and ophthalmoscopy.

**Figure 2:**
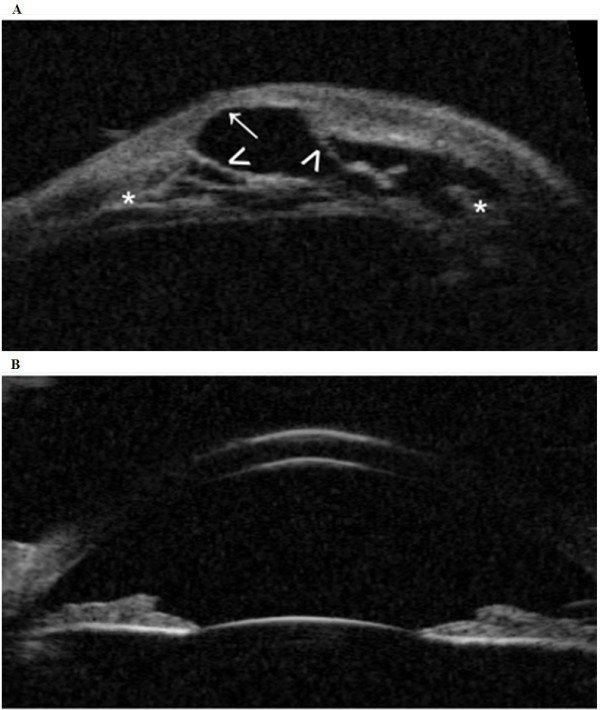
**Present an ultrasound biomicroscopy image (UBM) of both eyes taken with 50 MHz transducer (P60TM, Paradigm Medical Industries, Inc. Salt Lake City, UT). A** depicts the ultrasound biomicroscopy findings of the right eye: the transverse section in primary position shows irregularity of the corneal-depth with paracentral crater-like depression (arrow, ↑) involving the corneal endothelium and the Descemet’s membrane. The anterior chamber structures are hardly distinguishable (asterisk, *) showing kerato-irido-lenticular contact (open arrowheads, >). **B** shows the ultrasound biomicroscopy of the left eye, representing a normal, structured anterior segment.

On ultrasound biomicroscopy (UBM), anterior chamber structures of the right eye were difficult to differentiate, showing adhesions from the opacified cornea to the iris with a kerato-irido-lenticular contact to the remnant lens. No iridotrabecular angle could be differentiated. The central corneal defect was hyperechogenic and showed irregularity in deepness with a paracentral crater-like depression of the posterior cornea (Figure 2A). The contralateral eye showed a normal structure of the anterior segment (Figure 2B). Thus, the UBM findings pointed toward a unilateral anterior segment dysgenesis (ASD) in a form of Peters’ anomaly. Ophthalmic examinations, as well as chromosome analyses of the parents, including FISH, evaluated with the same probe as the child, were normal.

## Discussion

In the presented case, the clinical features such as elfin face, muscular hypotonia, patent foramen ovale and patent ductus arteriosus, mild mental retardation, as well as the peculiar behavior, suggested the diagnosis of WBS.

The previously reported ocular findings in this patient with WBS included a characteristic stellate pattern of the anterior iris stroma and retinal vascular tortuosity in the left eye. The novel findings were a microphthalmia and an ASD in a form of Peters’ anomaly in the right eye. Consistent with WBS, a deletion was found in the 7q11.23 region. It is not clear however, if there is an association between the deletion in the 7q11.23 region and the described unilateral ASD. While so far no genes in the deleted WBS region are known to affect the anterior segment, most genes in the 7q11.23 region are poorly understood, with the exception of *ELN*. As a result of mutation of one of the *ELN* alleles, a genetic link between infantile esotropia and WBS has already been hypothesized [8,20]. Identified to cause connective-tissue and vascular anomalies [14,21,22], the *ELN* has also been suggested to contribute to extraocular manifestations, such as the elfin face [4].

The *ELN* encodes elastin, a component of collagen tissue of the eye that is also present in the iris stoma [23]. Therefore, the stellate pattern of the iris stroma which is seen in almost 50% of WBS [24] could be hypothesized to be linked to the *ELN* deletion seen in our patient.

Although, an association between the haploinsufficiency of the *ELN* gene and the modification of corneal elasticity has been proposed, it seems unlikely as a histological observation has not confirmed the presence of elastin fibers in the examined corneas [25].

In our patient, using UBM, we were able to exclude the initially suspected corneal ectasia and confirm Peters' anomaly instead, as the paracentral crater-like depression involving the corneal endothelium and the Descemet’s membrane, showed a kerato-irido-lenticular contact. In ASD disorders, not only the iris, but also the ciliary body, anterior chamber, lens and cornea are involved. Here, even if the *ELN* deletion seems to be responsible for the stellate pattern, the *ELN* alone can not fully explain the Peters’ anomaly. In developmental phenotypes it is often difficult to rule out whether there are genetic predispositions that can result in unilaterality. Peters’ anomaly has not been reported in WBS patients so far. In WBS an association with another ASD, the Axenfeld-Rieger spectrum, has been described previously. In these patients the WBS was also associated with dental anomalies [10,26]. Although the *PITX2c* isoform has been identified to cause the right/ left asymmetry development [27], no *PITX2c* mutations have been reported in patients with Axenfeld-Rieger syndrome so far. Only an association of asymmetric ASD with dental malformation and brain retardation has been linked to the isoform *PITXc* expression [28]. In our case, although the ASD was associated with dental abnormalities, a search for *PITX2c* and related genes was not conducted.

Instead, in order to examine the influence of the deletion size on the unusual phenotype, we analyzed the deletion by microsatellite analysis. In the presented case the deletion size was found to be moderately large but not microscopically visible (length: 2.36 Mb), while the common deletions have a size of 1.29 Mb. The clinical phenotype appears to be more severely affected in cases with microscopically visible deletions, but not with larger submicroscopic deletions [29].

A combination of ASD and bilateral cleft of the soft and the hard palate, as in the presented patient, has not been linked to haploinsufficiency of a gene involved in the microdeletion. However, an association of a cleft palate with WBS has been described in two individual papers [30,31]. Pankow described monozygotic twins with WBS and a cleft palate. In this family the cleft palate was thought to be an autosomal dominant inheritance and thus independent of the WBS, as it occurred frequently on the father’s side of the family [31]. However, in the patient described by Blanco-Davila there was no previous family history of congenital malformations [30].

In our patient, the chromosome analyses of the parents were normal, but there was a positive family history for cleft palate. Therefore, while the association of cleft lip/palate anomaly in our patients is most likely related to positive family history, a possible connection between WBS and cleft palate cannot be ruled out.

## Conclusions

Unilateral Peters’ anomaly and cleft palate were diagnosed in a patient with classical sporadic Williams-Beuren syndrome. Even if a common cause of these conditions remains an intriguing speculation, a co-occurrence by chance is very likely. Arguments in favor of the latter are the unilaterality of the Peters’ anomaly, as well as, the fact that numerous patients with deletions of comparable or microscopically visible size have not shown similar manifestations.

### Consent

Written informed consent was obtained from the mother of the patient for publication of this Case Report and the accompanying images. A copy of the written consent is available for review by the Editor of the journal.

## Abbreviations

ASD: Anterior segment dysgenesis; ELN: Elastin; FISH: Fluorescence in-situ hybridization; Pitx2: Paired-like homeodomain transcription factor 2; UBM: Ultrasound biomicroscopy; WBS: Williams-Beuren syndrome.

## Competing interests

The authors declare that we have no competing interests.

## Authors’ contributions

MGT conceived of the study and is responsible for the content and for writing the paper. MG and MGT performed the UBM and collected the data. MG, PM and RJAC participated in the design of the study and helped to draft the manuscript. RJAC and PM preformed genetic analyses. MG, PM and AMPW critically revised the manuscript. All authors read and approved the final manuscript.

## Pre-publication history

The pre-publication history for this paper can be accessed here:

http://www.biomedcentral.com/1471-2415/14/70/prepub
